# Living with Epilepsy in Adolescence in Italy: Psychological and Behavioral Impact

**DOI:** 10.3390/healthcare11050687

**Published:** 2023-02-25

**Authors:** Katherine Turner, Francesca La Briola, Aglaia Vignoli, Elena Zambrelli, Valentina Chiesa, Laura Fongoni, Olivia Baldi, Maria Paola Canevini

**Affiliations:** 1 Epilepsy Center, Childhood and Adolescence Neuropsychiatry Unit, ASST Santi Paolo e Carlo, San Paolo Hospital, 20142 Milan, Italy; 2 Department of Health Sciences, University of Milan, 20142 Milan, Italy

**Keywords:** epilepsy, adolescence, behavioral disorders, psychiatric comorbidities

## Abstract

Background: People with epilepsy have a higher prevalence of behavioral and neuropsychiatric comorbidities compared to the general population and those with other chronic medical conditions, although the underlying clinical features remain unclear. The goal of the current study was to characterize behavioral profiles of adolescents with epilepsy, assess the presence of psychopathological disorders, and investigate the reciprocal interactions among epilepsy, psychological functioning, and their main clinical variables. Methods: Sixty-three adolescents with epilepsy were consecutively recruited at the Epilepsy Center, Childhood and Adolescence Neuropsychiatry Unit of Santi Paolo e Carlo hospital in Milan (five of them were excluded) and assessed with a specific questionnaire for psychopathology in adolescence, such as the Questionnaire for the Assessment of Psychopathology in Adolescence (Q-PAD). Q-PAD results were then correlated with the main clinical data. Results: 55.2% (32/58) of patients presented at least one emotional disturbance. Body dissatisfaction, anxiety, interpersonal conflicts, family problems, uncertainty about the future, and self-esteem/well-being disorders were frequently reported. Gender and poor control of seizures are associated with specific emotional features (*p* < 0.05). Conclusions: These findings highlight the importance of screening for emotional distress, recognition of the impairments, and provision of adequate treatment and follow-up. A pathological score on the Q-PAD should always require the clinician to investigate the presence of behavioral disorders and comorbidities in adolescents with epilepsy.

## 1. Introduction

Adolescents with epilepsy are at increased risk of developing psychological and behavioral disorders compared to the general population and those with other chronic medical conditions [1,2].

The International League Against Epilepsy (ILAE) has identified that the prevalence of neuropsychiatric disorders in children with epilepsy is around 35–50%, and over 50% presented an intellectual disability (ID) [3].

Several investigations have reported that up to 50% of subjects go undetected and untreated. Mood, anxiety, and attention-deficit hyperactivity disorder (ADHD) occur most frequently in adolescents with epilepsy [4,5].

Neuropsychiatric symptomatology in pediatric epilepsy has been associated with different hypotheses. Firstly, the unpredictable nature of seizures could influence psychological development, thereby fostering psychiatric disorders. Moreover, coexisting neurological comorbidities may contribute to the development of psychological distress.

Several authors have found that ID is the most common neuropsychiatric manifestation in children with epilepsy (30–40%) [6,7].

Of note, anti-seizure medications may cause cognitive and behavioral side effects. Furthermore, epileptogenic circuits could be linked with modifications in brain structure and function, possibly resulting, through many different mechanisms, in neuropsychiatric manifestations [8].

Behavioral disturbances can precede epilepsy onset or may be present in the early phase; indeed, the association between epilepsy and behavioral difficulties can be bidirectional [9]. Some authors have suggested that depressed mood and anxiety may precede the onset of epilepsy and act as independent risk factors for the development of unprovoked seizures [10,11].

The worldwide pandemic caused by the SARS-CoV-2 virus was associated with an increased risk of psychiatric sequelae in the general population [12]. Furthermore, during the pandemic, a significant number of patients with epilepsy experienced worsening psychological symptoms and an increase in seizure frequency [13,14].

Within this perspective, in the present study, we investigated the psychological and behavioral sequelae experienced by a group of adolescents with epilepsy using a specific questionnaire for psychopathology in adolescence, the Assessment of Psychopathology in Adolescence (Q-PAD). We also investigated whether the clinical characteristics of epilepsy, such as epilepsy type, epilepsy onset, seizure frequency, anti-seizure medications, age, intelligence quotient (IQ), and gender interfere with emotional well-being.

## 2. Materials and Methods

### 2.1. Participants

Patients with a diagnosis of epilepsy were consecutively recruited and prospectively evaluated within this study.

The inclusion criteria included: (1) diagnosis of epilepsy; (2) aged between 14 and 19 years old; and (3) fluency in the Italian language. The exclusion criteria were: (1) intellectual disability; (2) major progressive neurological pathologies; (3) history of psychiatric disorders (diagnosis according to DSM-5); and (4) receiving medications other than antiepileptic drugs. We included patients with a total IQ under the normal range but with a normal General Ability Index (GAI). It is well known that patients with epilepsy have more difficulty in working memory and processing speed, so the GAI could be considered a more accurate representation of their intelligence.

The diagnosis of epilepsy was based on the ILAE classification [15]. Seizure types were classified according to clinical semiology provided by description from a witness or, when possible, by electroencephalogram (EEG) or video–EEG recording of the episode [16]. Patients were considered seizure-free if they were without clinical seizures for at least 1 year, using the last clinical visit documenting seizure status as the end point of follow-up. Refractory epilepsy was defined as uncontrolled seizures after at least two first-line anticonvulsant medication trials [17]. Intelligence assessments were performed according to best practice standards using the Wechsler Intelligence Scale for Children (WISC-III or WISC-IV) or Wechsler Adult Intelligence Scale (WAIS-R or WAIS-IV). For all patients, intelligence assessments were selected according to best practice standards. We classified patient IQ (intelligence quotient) as follows: normal IQ; borderline intellectual functioning (BIF); and mild, moderate, severe or profound ID according to Diagnostic and Statistical Manual of Mental Disorders, Fifth Edition (DSM-5) criteria [18].

The sample included 63 adolescents attending the Epilepsy Center, Childhood and Adolescence Neuropsychiatry Unit of Santi Paolo e Carlo Hospital, University of Milan, Milan. Two adolescents refused to participate in the study.

We enrolled our patients over a span of two years (2019–2020).

All procedures performed in studies involving human participants were in accordance with the ethical standards of the institutional and/or national research committee and with the 1964 Helsinki declaration and its later amendments or comparable ethical standards. The Ethics Committee of San Paolo Hospital reviewed and approved the study protocol.

All patients and their caregivers provided their informed written consent before being enrolled in the study.

### 2.2. Questionnaire

The Questionnaire for the Assessment of Psychopathology in Adolescence (Q-PAD) is a self-administered instrument to assess psychopathological domains described in the scientific literature regarding adolescents aged from 14 to 19 years. The questionnaire contains 81 items in a Likert-type scale with values from 1 to 4, formulated in a language that is familiar according to the linguistic uses of adolescents. The scores of each item are added up for each scale and then converted to percentile values. Therefore, the output of the Q-PAD consists of eight scores referring to the following main psychological areas: body dissatisfaction, anxiety, depression, substance abuse, interpersonal conflicts, family problems, uncertainty about the future, and psychosocial risks. A further ninth domain, called self-esteem and well-being, concerns well-being and adjustment and is assessed in positive terms. The questionnaire has been validated on a sample of 1454 Italian adolescents (Internal Consistency: 0.83, Test-Retest Reliability: 0.84) [19].

### 2.3. Data Analysis

All statistical analyses were performed using the Statistical Package for Social Sciences (SPSS) version 27.0 for Windows. Qualitative data were reported as absolute frequencies and percentages. Quantitative data were expressed as means and standard deviations (SDs).

Demographics and clinical variables of adolescents were compared with the Mann–Whitney *U* test or Student’s *t*-Test for independent samples, depending on the distribution. The normality of the data and homogeneity of variances were tested by the Shapiro–Wilk test and Levene’s test, respectively.

Significance was set at a *p*-value of 0.05.

We compared the questionnaires by dividing the participants according to the type of epilepsy, epilepsy onset, frequency of seizures, controlled seizures vs. uncontrolled, anti-seizure medications, age (age 14–15 years vs. 16–19 years), IQ, and gender.

## 3. Results

### 3.1. Patient Characteristics and Clinical Variables

Sixty-three patients (mean age of 17.24 ± 2.05 years, range: 14–19 years; 19 [32.8%] males, 39 [67.2%] females) were consecutively enrolled in the study; five of them had to be excluded for the following reasons: two showed moderate–severe ID, and three patients were not Italian native speakers (two Hispanic and one Arabic).

Demographic and clinical data were collected from the patients’ medical charts.

Forty (69.0%) patients had focal epilepsy (23 [39.7%] focal epilepsy of unknown etiology, 17 [29.3%] structural focal epilepsy); 18 patients (31.0%) had generalized genetic epilepsy (GGE).

The mean age of epilepsy onset was 10.05 years (±5.1), and the mean duration since diagnosis was 8.07 years (±5.82).

Characteristics of patients are presented in Table 1.

### 3.2. Psychological Variables

**Figure 1:**
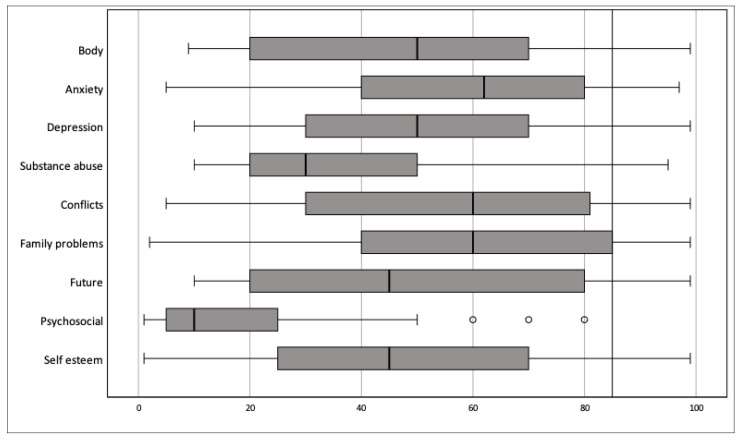
Distribution of adolescents with epilepsy on the Q-PAD. Figure legend: distribution of Q-PAD scores (minimum score, first (left) quartile, median, third (right) quartile, and maximum score), ° outlier value. Abbreviations: Q-PAD: Questionnaire for the Assessment of Psychopathology in Adolescence.

The Q-PAD findings suggest the presence of at least one neuropsychiatric disturbance in 55.2% (32/58) of the adolescents with epilepsy. Our sample had a clinical range of body dissatisfaction (19.0%), anxiety (19.0%), depression (6.9%), substance abuse (1.7%), interpersonal conflicts (22.4%), family problems (25.9%), and uncertainly about the future (22.4%). Moreover, concerning self-esteem/well-being, 31.0% had a normal range, 20.7% borderline values and 48.3% showed a clinical range (Figure 1, Table 2).

Statistically significant differences were found considering age as a covariate: older adolescents (age 16–19 years) showed more interpersonal conflicts (mean = 63.5 ± 26.86 vs. mean = 42.89 ± 30.0) and uncertainty about the future (mean = 55.77 ± 29.27 vs. mean = 37.39 ± 27.16) than younger patients (age 13–15 years; *p* = 0.016, *p* = 0.022 respectively).

### 3.3. Clinical Variables x Psychological Variables

**Figure 2:**
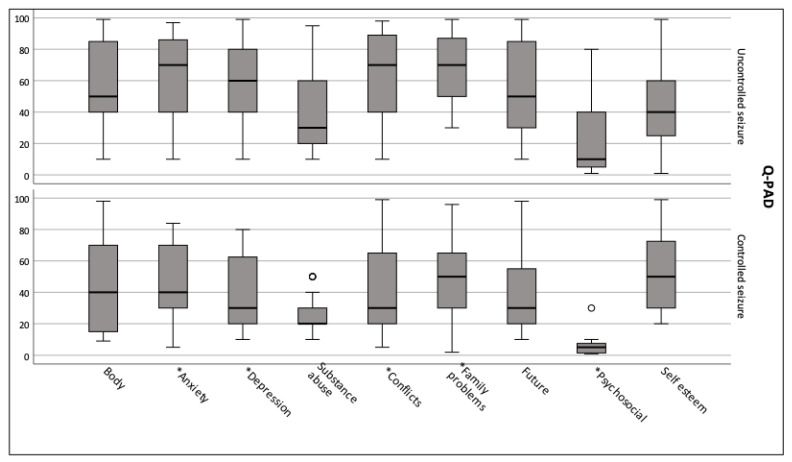
Q-PAD scores for adolescents with uncontrolled seizures vs. controlled seizures. Figure legend: Q-PAD scores of adolescents with uncontrolled seizures compared to patients with controlled seizures (minimum score, first (lower) quartile, median, third (upper) quartile, and maximum score). * Significant scores. ° Outlier value. Abbreviations: Q-PAD: Questionnaire for the Assessment of Psychopathology in Adolescence.

We evaluated the association between some of the clinical variables and the psychological constructs. The type and the onset of epilepsy had no impact on Q-PAD measures. However, we found a statistically significant difference between adolescents with controlled seizures vs. uncontrolled seizures on anxiety, depression, interpersonal conflicts, family problems, and psychosocial risks (*p* < 0.05, Figure 2).

In relation to treatment (monotherapy vs. polytherapy) and IQ (Normal IQ vs. Borderline Intellectual Functioning), we found no differences in the psychological dimensions investigated (*p* > 0.05). Finally, with respect to gender, we found no differences in the psychological dimensions investigated apart from depression; girls tended to be more depressed (mean = 54.74 ± 27.77) than boys (mean = 37.89 ± 24.39, *p* = 0. 027).

## 4. Discussion

Although most studies on epilepsy and psychological variables have gathered information from parents and teachers, an interesting aspect of our work was to focus on the patient’s point of view. As a general result, our data showed that adolescents with epilepsy have a high rate of neuropsychiatric disturbances (55.2%, 32/58), in accordance with the literature [20,21]. We found a clinical range of body dissatisfaction (19.0%), anxiety (19.0%), depression (6.9%), substance abuse (1.7%), interpersonal conflicts (22.4%), family problems (25.9%), uncertainly about the future (22.4%), and low self-esteem/well-being (48.3%). These findings are thus in accordance with a previous meta-analysis by Lin and colleagues, showing that subjects with epilepsy are at increased risk for neuropsychiatric disorders, such as internalizing behaviors (anxiety, mood and social disorders) prevailing over externalizing functioning (aggressive outbursts or conduct disorders) [20]. While these manifestations may be considered consequences of epilepsy, increasing evidence suggests that these disturbances might precede the onset of epilepsy and seizures. Therefore, epilepsy and neuropsychiatric issues may have a bidirectional correlation, sharing a common underlying pathogenesis [10,11].

Psychiatric disorders, in particular mood and anxiety disorders, may be a reaction to psychosocial obstacles, lower quality of life, and perceived stigmatization by parents [11]. Providing patients, especially those with drug-resistant seizures, with psychological support to orientate their psychological patterns to efficacious ones, improve the overall quality of life and emotional well-being, and reduce fatigue, should be seriously considered. Such cognitive–behavioral programs could help adolescents to adopt more effective ways of coping with their clinical conditions and experience easier psychosocial adjustment [22,23]. Moreover, we found that older adolescents (aged from 16 to 19 years) have more interpersonal conflicts and uncertainty about the future compared to younger adolescents (aged from 14 to 15 years); this result is consistent with previous studies [24].

Considering the clinical variables, our results showed that the type of epilepsy, anti-seizure medication, epilepsy onset, epilepsy duration, and IQ do not influence the answers to the questionnaire.

The scores on the Q-PAD were markedly higher in people with refractory epilepsy, which represents, therefore, one of the major risk factors for poor mental health in childhood.

Adolescents with active epilepsy are at higher risk for anxiety, mood disorders, interpersonal conflicts, and family problems and have higher psychosocial risk. All these outcomes should be closely followed up to identify a possible pathway away from stigma and loneliness as early as possible [25].

Some authors have suggested that higher rates of anxiety and mood disorders are associated with the occurrence of seizures in public places [26].

Davies et al. reported that both uncomplicated and complicated epilepsy groups showed a substantial increase in emotional and behavioral disorders. However, only the complicated epilepsy group (identified as having additional neurological problems or severe learning difficulties) was associated with a markedly increased rate of hyperactive and pervasive developmental disorders [27].

Adolescence is characterized by physical, psychological, and emotional changes. Young people may feel intense emotions, fear for the future, low self-esteem, and difficulties with peers. Epilepsy has an important psychological and social impact on adolescents at this critical time of life [28]. The fact that mental health disturbances are much more commonly associated with epilepsy than with other chronic pathologies, such as diabetes or asthma, indicates that the psychological consequences of epilepsy are not an inevitable result of a chronic and potentially life-threatening disease that requires daily therapy; neurological pathology and social stigma are likely to be key risk factors [27,29].

It is well known that female patients may suffer from mood disorders 2–3 times more often than males; our data are in accordance with the findings that the female gender had an increased incidence of depressive disorders compared to males [30,31].

The early identification and appropriate management of these disturbances should translate into better seizure control, fewer anti-seizure medications and side effects, improved quality of life, reduced costs of healthcare delivery, and better outcomes for society as a whole [3,27,32].

The COVID-19 epidemic increased the prevalence of mental health problems such as depression, anxiety, and sleep disorders in people with epilepsy [33]. During the unprecedented lockdown in Italy, many patients with chronic conditions lost their regular follow-up programs; for this reason, it is crucial to monitor the impact of COVID-19 on this vulnerable cohort [34].

This research has an important strength: to our knowledge, this is the first work to investigate the influence of behavioral disorders in adolescents with epilepsy by focusing on the patient’s point of view and perspective.

Our work has some limitations to disclose. Firstly, the study was conducted in a single center, reflecting the practice style of our tertiary Epilepsy Center, and the subjects were referred to our epilepsy center for pharmacoresistant epilepsy. Secondly, we did not have any controls with another chronic pathology to verify whether they demonstrated similar psychiatric profiles. Thirdly, the study was questionnaire-based, and an in-depth clinical psychiatric interview with a specific focus on psychological/psychiatric problems was missing, in particular concerning the assessment of disease severity.

## 5. Conclusions

Adolescents with epilepsy exhibit specific psychobiological profiles. Future research is warranted to evaluate the implications from both a research perspective (the genes and circuits implicated) and from a clinical perspective. Our results encourage physicians to always explore psychological issues in adolescents with epilepsy and suggest potential areas of intervention. In conclusion, the psychological assessment of adolescents affected by epilepsy should be integrated with clinical practice to promote early diagnosis and management, considering the higher prevalence of behavioral disturbances in this population.

## Figures and Tables

**Table 1:** Demographic and clinical characteristics.

Variable	N = 58
Age (years)	
Mean	17.24
SD	2.05
Range	14–19
Younger Adolescents (14–15 years)	18 (31.0%)
Older Adolescents (16–19 years)	40 (69.0%)
Gender	
Male	19 (32.8%)
Female	39 (67.2%)
Education (years)	
Mean	10.36
SD	2.13
Range	7–13
Type of epilepsy	
Focal Epilepsy	
Unknown	23 (39.7%)
Structural	17 (29.3%)
Generalized Genetic Epilepsy	18 (31.0%)
Epilepsy onset (years)	
Mean	10.05
SD	5.1
Range	0–18
Epilepsy duration (years)	
Mean	8.07
SD	5.82
Range	0-28
Anti-seizure Medications	
Monotherapy	26 (44.8%)
Polytherapy	23 (39.7%)
No anti-seizure Medications	9 (15.5%)
Seizure frequency	
Daily	3 (5.2%)
Weekly	5 (8.6%)
Monthly	4 (6.9%)
Sporadic	15 (25.9%)
None	31 (53.4%)
Intelligence assessment	
Total IQ	
Mean	86.50
SD	23.69
Verbal IQ	
Mean	80.66
SD	25.67
Performance IQ	
Mean	82.90
SD	20.80
Verbal Comprehension	
Mean	100.33
SD	14.54
Perceptual Reasoning	
Mean	113.56
SD	12.32
Working Memory	
Mean	96.89
SD	15.10
Processing Speed	
Mean	87.78
SD	14.63

Abbreviations: SD: standard deviation; IQ: intelligence quotient.

**Table 2:** Q-PAD scores.

Q-PAD Scores	
Variable	N = 58
Body dissatisfaction	
Mean	51.59
SD	28.87
Range	9–99
Normal range	47 (81.03%)
Clinical range	11 (18.97%)
Anxiety	
Mean	57.16
SD	26.84
Range	5–97
Normal range	47 (81.03%)
Clinical range	11 (18.97%)
Depression	
Mean	49.22
SD	27.67
Range	10–99
Normal range	54 (93.10%)
Clinical range	4 (6.90%)
Substance abuse	
Mean	34.47
SD	21.85
Range	10–95
Normal range	57 (98.28%)
Clinical range	1 (1.72%)
Interpersonal conflicts	
Mean	57.10
SD	29.23
Range	5–99
Normal range	45 (77.6%)
Clinical range	13 (22.4%)
Family problems	
Mean	59.71
SD	25.67
Range	2–99
Normal range	43 (74.1%)
Clinical range	15 (25.9%)
Uncertainty about the future	
Mean	50.07
SD	29.66
Range	10–99
Normal range	45 (77.6%)
Clinical range	13 (22.4%)
Psychosocial risk	
Mean	16.44
SD	19.49
Min-Max	1–80
Normal range	58 (100%)
Self-esteem/well-being	
Mean	48.58
SD	27.88
Range	1–99
Normal range	18 (31.6%)
Borderline	12 (21.0%)
Clinical range	27 (47.4%)

Abbreviations: SD: standard deviation; Q-PAD: Questionnaire for the Assessment of Psychopathology in Adolescence.

## Data Availability

The data presented in this study are available upon request from the corresponding author.
